# Malignant tumours of temporomandibular joint

**DOI:** 10.1186/s12885-020-07425-9

**Published:** 2020-10-06

**Authors:** Feiluore Yibulayin, Chen-xi Yu, Lei Feng, Meng Wang, Meng-meng Lu, Yuan Luo, Hui Liu, Zhi-cheng Yang, Alimujiang Wushou

**Affiliations:** 1 Department of Oral & Maxillofacial Surgery and Oral Biomedical Engineering Laboratory Shanghai Stomatological Hospital Fudan University, 356 Beijing East Road, Shanghai, 200001 People’s Republic of China; 2 Department of Preventive Medicine, School of Public Health, Shanghai Medical College, Fudan University, Shanghai, 200001 People’s Republic of China; 3 Department of Clinical Medicine, Shanghai Medical College, Fudan University, Shanghai, 200001 People’s Republic of China

**Keywords:** Temporomandibular joint, Malignant tumour, SEER analysis

## Abstract

**Background:**

Malignant tumours of the temporomandibular joint (MTTMJ) are extremely rare. Studies describing its unique epidemiology, clinicopathological features, treatment and prognosis comprehensively are limited. To address these issues, current investigation was performed.

**Methods:**

A retrospective research was carried out by using population-based data from the Surveillance, Epidemiology, and End Results database (1973–2016).

**Results:**

Data for a total of 734 patients, including 376 men and 358 women, was found. The median age was 47 years. The 5-year and 10-year disease specific survival (DSS) rates were 69.2 and 63.6%, respectively. Significant differences in DSS were found according to age, race, tumour type, AJCC/TNM stage, surgery, radiotherapy, chemotherapy and different treatment modalities (*P* < 0.05). In the multivariate survival analysis, age > 44 years and AJCC stage III and IV were associated with poor DSS.

**Conclusion:**

MTTMJ was mostly found in white people with a median age of 47 years without any sex predominance. Patient’s age and AJCC stage was independent predictor of DSS.

## Background

Temporomandibular joint (TMJ) disorders are very common and can be easily diagnosed [1, 2]. However, malignant tumours of the temporomandibular joint (MTTMJ) are very rare and often cause facial asymmetry deformity and occlusal disorders [3]. MTTMJ originates from three possible sites: (a) intrinsic tissue of the TMJ, (b) extension of malignant tumours from adjacent issues, such as parotid gland malignant neoplasm, and (c) distant metastatic spread to the joint. Among these tumours, primary tumours from intrinsic tissue of the TMJ are extremely rare. MTTMJ patients may present with complex signs and symptoms that mimic those of myofascial pain and dysfunction syndrome, such as TMJ disorders [4]. As a result, the clinical manifestation and differential diagnosis of TMJ malignancies is challenging for primary care doctors [5].

Early diagnosis and treatment are important in achieving good prognosis in MTTMJ patients. As it is a solid tumour, surgical resection is the most important treatment modality for MTTMJ, and chemoradiotherapy is given as adjuvant therapy for advanced stage tumours and metastatic disease [6]. Surgical reconstruction of the TMJ is difficult, and poor treatment could result in loss of function, disfigurement, occlusal disorders and psychosocial morbidities [7]. To date, there are over 1000 reports regarding TMJ malignancy in PubMed [8]. However, case reports and reviews accounted for the majority of these studies [9, 10].

Because MTTMJ is rare, there is lack of instructive data to characterize its unique epidemiology, clinicopathological features, treatment and prognosis comprehensively. A nationwide population-based cohort may provide an opportunity to address these issues. Thus, we performed current retrospective analysis by using data from the Surveillance, Epidemiology, and End Results (SEER) database (1973–2016).

## Methods

### Data extraction

SEER*Stat software was applied for data extraction (https://seer.cancer.gov, version 8.3.6). Primary MTTMJ cases were identified by using International Classification of Diseases for Oncology (ICD-O-3) topographic codes C41.1-mandible with bones and joints. The variables for analysis included pathological tumour types, age at diagnosis, sex, race, pathological differentiation, American Joint Committee on Cancer (AJCC) stage, treatment modalities, vital status and follow-up time. Our study used established data and did not involve interactions with human subjects. Therefore, institutional review board approval was not required.

### Statistical analysis

Statistical analysis was performed by using the statistical packages R (The R foundation, http://www.r-project.org, version 3.4.3), Empower R (http://www.empowerstats.com, Boston, Massachusetts), and Statistical Package for Social Sciences (SPSS, Chicago, IL, Version 23.0 for Windows). Student’s t-test or the non-parametric Wilcoxon test were used for numerical variable evaluation, and the categorical variables were compared with the chi-square test or Fisher’s exact test. Kaplan-Meier survival analysis was used to assess overall survival (OS) and disease-specific survival (DSS). Prognostic factors were determined using the Cox multivariant regression model. *P* values were considered statistically significant when *P* < 0.05.

## Results

### Summary statistics for the total study population

**Fig. 1:**
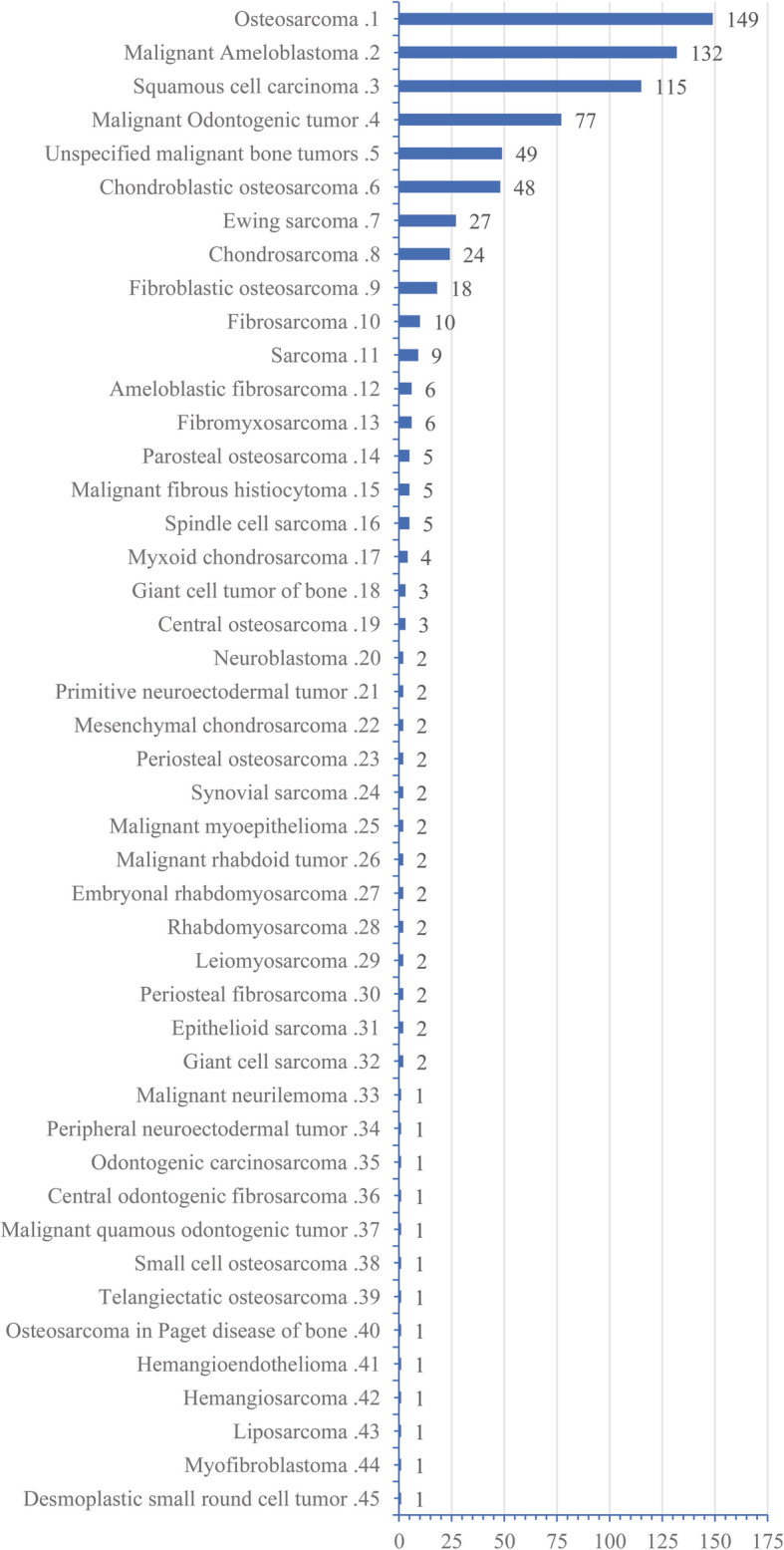
The tumor types of TMJ registered in the SEER database

A total of 734 primary cases were identified in the SEER database. The sex distribution was nearly equal with 376 males and 358 females. The median follow-up time period was 58 months (range, 0–499 months). MTTMJ mostly occurred in white people (70.3%, 516/734). MTTMJ was distributed across all ages, and the median age was 47 years. There were more than 40 different pathological tumour types (Fig. 1). The top three pathological types were osteosarcoma (149 cases), malignant ameloblastoma (132 cases) and squamous cell carcinoma (115 cases). Surgery was the mainstay treatment, and 562 patients received surgery. The summary of the study cohort’s clinicopathologic characteristics is presented in Table 1.

**Table 1:** The summary of MTTMJ patients’ clinico-pathologic characteristics

Variables	Disease specific survival (***n*** = 611)			Overall survival (***n*** = 734)		
	Alive (%)	Dead (%)	***P***-value	Alive (%)	Dead (%)	***P***-value
**Gender**						
Female	198 (64.7%)	108 (35.3%)	0.67	199 (55.6%)	159 (44.4%)	0.711
Male	203 (66.6%)	102 (33.4%)		203 (54%)	173 (46%)	
**Age**						
≤ 44 (47)	272 (81%)	64 (19%)	0.000	272 (74.9%)	91 (25.1%)	0.000
> 44 (47)	129 (46.9%)	146 (53.1%)		130 (35%)	241 (65%)	
**Age period**						
0–19	84 (83.2%)	17 (16.8%)	0.000	84 (77.1%)	25 (22.9%)	0.000
20–29	78 (83.9%)	15 (16.1%)		78 (79.6%)	20 (20.4%)	
30–39	69 (80.2%)	17 (19.8%)		69 (74.2%)	24 (25.8%)	
40–49	55 (75.3%)	18 (24.7%)		55 (65.5%)	29 (34.5%)	
50–59	55 (66.3%)	28 (33.7%)		55 (36.4%)	46 (45.5%)	
60–69	36 (49.3%)	37 (50.7%)		36 (36.4%)	63 (63.6%)	
70–79	17 (28.3%)	43 (71.7%)		18 (22%)	64 (78%)	
80+	7 (2.9%)	35 (97.1%)		7 (10.3%)	61 (89.7%)	
**Race**						
Others	59 (79.7%)	15 (20.3%)	0.001	59 (71.1%)	24 (28.9%)	0.000
Black	88 (73.9%)	31 (26.1%)		88 (65.2%)	47 (34.8%)	
White	254 (60.8%)	164 (39.1%)		255 (49.4%)	261 (50.6%)	
**Pathological grade**						
Grade I	37 (38.5%)	17 (31.5%)	0.140	37 (56.1%)	29 (43.9%)	0.318
Grade II	70 (69.3%)	31 (30.7%)		71 (56.8%)	54 (43.2%)	
Grade III	40 (53.3%)	35 (46.7%)		40 (45.5%)	48 (54.5%)	
Grade IV	45 (65.2%)	24 (34.8%)		45 (57.7%)	33 (42.3%)	
**AJCC Stage**						
Stage I	59 (92.2%)	5 (7.8%)	0.000	59 (86.8%)	9 (13.2%)	0.000
Stage II	54 (74.0%)	19 (77.8%)		54 (65.1%)	29 (34.9%)	
Stage III	2 (22.2%)	7 (77.8%)		2 (20%)	8 (80%)	
Stage IV	6 (66.7%)	3 (33.3%)		6 (54.5%)	5 (45.5%)	
**T stage**						
T1	33 (73.3%)	12 (26.7%)	0.324	33 (66%)	17 (34%)	0.721
T2	121 (81.8%)	27 (18.2%)		121 (72.9%)	45 (27.1%)	
T3	14 (70%)	6 (30%)		14 (70%)	6 (30%)	
T4	1 (50%)	1 (50%)		1 (50%)	1 (50%)	
**N stage**						
N0	155 (82%)	34 (18%)	0.001	155 (74.2%)	54 (25.8%)	0.006
N1	5 (38.5%)	8 (61.5%)		5 (35.7%)	9 (64.3%)	
NX	9 (69.2%)	4 (30.8%)		9 (60%)	6 (60%)	
**M stage**						
M0	163 (81.1%)	38 (18.9%)	0.003	163 (74.1%)	57 (25.9%)	0.001
M1	3 (42.9%)	4 (57.1%)		3 (30%)	7 (70%)	
MX	3 (42.9%)	4 (57.1%)		3 (37.5%)	5 (62.5%)	
**Surgery**						
No	31 (40.8%)	45 (59.2%)	0.000	31 (33.7%)	61 (66.3%)	0.000
Yes	352 (74.9%)	118 (25.1%)		353 (62.8%)	209 (37.2%)	
**Radiotherapy**						
No	305 (70%)	131 (30%)	0.000	305 (57.3%)	227 (42.7%)	0.005
Yes	86 (53.1%)	76 (46.9%)		84 (45.4%)	101 (54.6%)	
**Chemotherapy**						
No	295 (67.5%)	142 (32.5%)	0.122	295 (55.2%)	239 (44.8%)	0.673
Yes	106 (60.9%)	68 (39.1%)		107 (53.5%)	93 (46.5%)	

### OS analysis

**Fig. 2:**
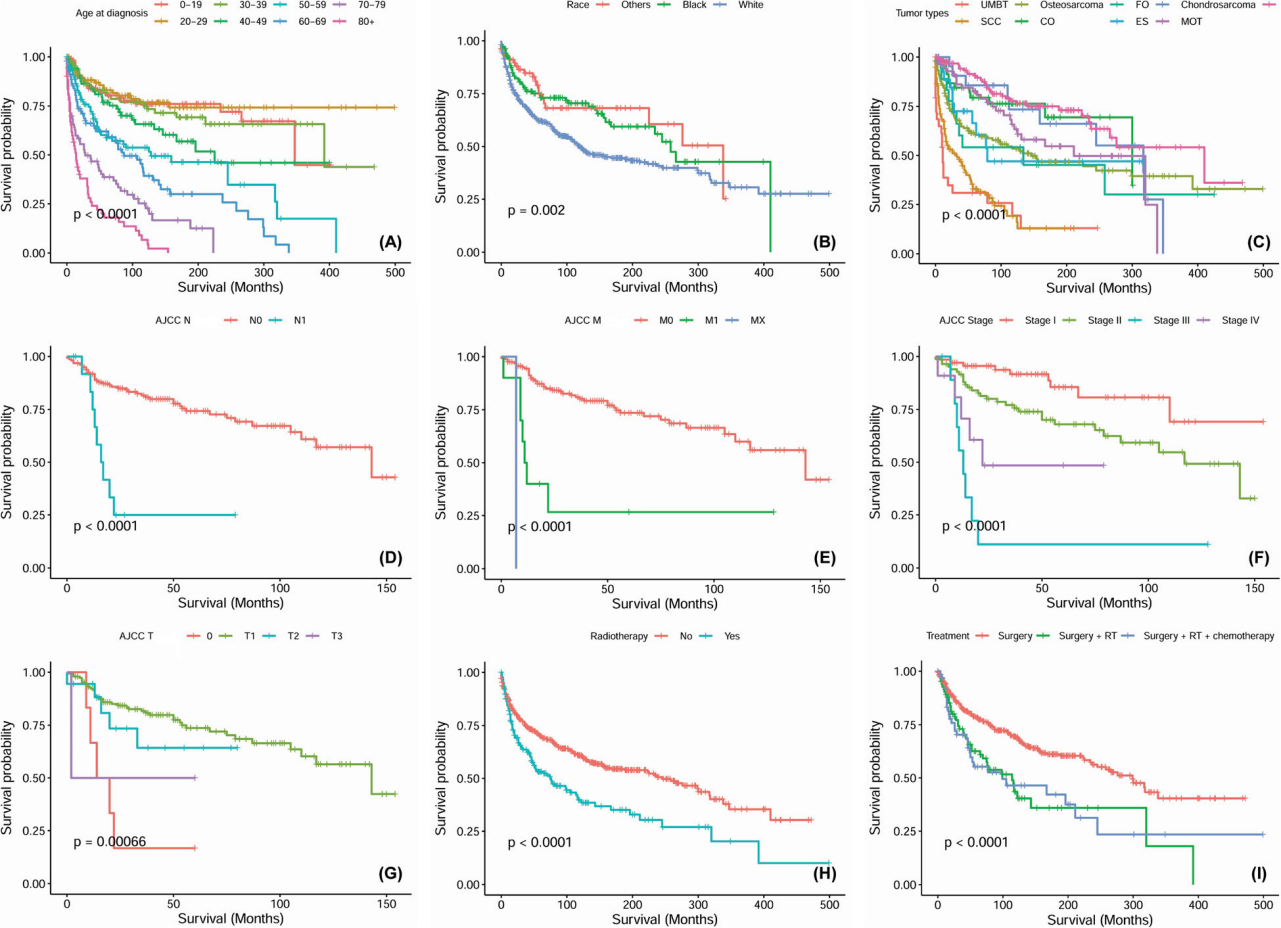
Overall survival curves of cases with MTTMJ compared according to (**a**) age range, (**b**) race, (**c**) tumor types, (**d**) AJCC N category, (**e**) AJCC M category, (**f**) AJCC stage, (**g**), AJCC T category, (**h**) radiotherapy, (**i**) different treatment modalities. *Abbreviation*- UMBT: unspecified malignant bone tumors, OS: osteosarcoma, FO: fibroblastic osteosarcoma, CS: Chondrosarcoma, MA: malignant ameloblastoma, SCC: squamous cell carcinoma, CO: chondroblastic osteosarcoma, ES: Ewing sarcoma, MOT: malignant Odontogenic tumor

The 3-year, 5-year and 10-year OS rates were 72, 65 and 55%, respectively. The 5-year OS was 78.4% for patients treated by surgery only, 61% for those who received both surgery and radiotherapy and 54% for others combined with surgery and chemoradiotherapy. Significant OS differences were identified depending on age at diagnosis (*P <* 0.0001), race (*P =* 0.002), tumour type (*P <* 0.0001), AJCC T category (*P =* 0.0007), AJCC N category (*P <* 0.0001), AJCC M category (*P <* 0.0001), AJCC stage (*P <* 0.0001) and treatment modality (*P <* 0.0001) (Fig. 2). The Cox proportional hazards regression model was constructed to evaluate predictors of OS via multivariate survival analysis. Age > 47 years [HR (95% CI) = 2.76 (1.15–6.65), *P* = 0.024, age ≤ 47 years as the reference value] and AJCC M1 category [HR (95% CI) = 36.91(5.58–118.35), *P* = 0.024, AJCC M0 stage as the reference value] were independently associated with worse OS.

### DSS analysis

**Fig. 3:**
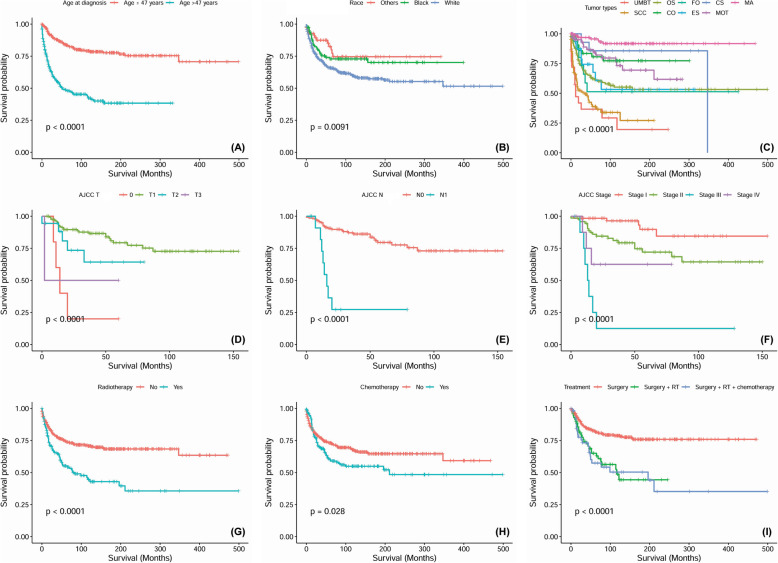
Disease specific survival curves of cases with MTTMJ compared according to (**a**) age at diagnosis, (**b**) race, (**c**) tumor types, (**d**) AJCC T category, (**e**) AJCC N category, (**f**) AJCC stage, (**g**) radiotherapy, (**h**) chemotherapy and (**i**) different treatment modalities. Log-rank test was utilized to compare curves, and significance is presented on each panel. *Abbreviation*- UMBT: unspecified malignant bone tumors, OS: osteosarcoma, FO: fibroblastic osteosarcoma, CS: Chondrosarcoma, MA: malignant ameloblastoma, SCC: squamous cell carcinoma, CO: chondroblastic osteosarcoma, ES: Ewing sarcoma, MOT: malignant Odontogenic tumor

In the survival analysis for DSS, the 3-year, 5-year and 10-year DSS rates were 74.7, 69.2 and 63.6%, respectively. Similarly, patients who received surgical treatment only had an 82% 5-year DSS rate; the 5-year DSS rate was 63% for those who received surgery plus radiotherapy and 55.3% for those who received surgery and chemoradiotherapy. Statistically significant survival differences were found among different treatment modalities (*P <* 0.0001). We also identified significant differences in DDS based on age range at diagnosis (*P <* 0.0001), median age (*P <* 0.0001), race (*P =* 0.0091), pathological tumour type (*P <* 0.0001), AJCC T category (*P <* 0.0001), AJCC N category (*P <* 0.0001), AJCC M category (*P <* 0.0001), AJCC stage (*P <* 0.0001), surgery (*P <* 0.0001), radiotherapy (*P <* 0.0001) and chemotherapy (*P =* 0.0028) (Fig. 3). Age > 44 years [HR (95% CI) = 2.72 (1.23–5.97), *P* = 0.013, age ≤ 44 years as the reference value] and AJCC stage III and IV [HR (95% CI) = 19.85 (5.6–70.34), *P <* 0.0001; HR (95% CI) = 7.1 (1.34–737.62), *P* = 0.021, AJCC stage I as the reference value] were adversely associated with DSS.

## Discussion

Apart from single case reports and small retrospective case series, there are insufficient data to characterize the demographic features of MTTMJ. Generally, the average age range of most patients with solid malignancies in the head and neck is 60–70 years [11]. The typical incidence of MTTMJ occurred in the age range of 40–60 years old in most previous reports [7, 12–15]. The current study results show that the MTTMJ was almost evenly distributed among all age groups, with a median age of 47 years, and the sex ratio of males to females was close to 1:1. Previous MTTMJ studies were mainly based on a single center’s institutional experience. The biggest disadvantage of these studies is that the sample size is too small. Thus, it is impossible to perform epidemiologically relevant survival analysis. Here, we performed the first survival analysis regarding the age, sex and race of MTTMJ patients and found survival differences between these variables. Most importantly, we determined patient age as an independent prognostic factor for DSS and OS.

TNM/AJCC staging plays an irreplaceable role in head and neck cancer treatment. It is helpful to oncologists in determining treatment protocols and cancer prognosis [16]. However, in any previous studies, the TNM/AJCC staging was not used to evaluate the prognosis of MTTMJ. Most of the data on TNM staging are missing in our study. We evaluated survival using the available data, and typical differences were identified between these parameters. Among them, M category and AJCC stage were independently associated with DSS and OS. However, the conclusion is not very convincing. There are some confounding factors. First, the exact origin of the tumour is unknown. Second, all tumour types were analysed in a mixed fashion. These facts possibly weaken the conclusion since tumours of different origins and types have different biological behaviours and prognosis.

Surgical resection and reconstruction are the most important mainstay treatment modalities for solid tumours in the oral and maxillofacial region. Treatment information was missing in 172 patients, and the rest underwent surgery. The prognosis of patients treated with surgery alone was better than that of patients treated with surgery combined with chemoradiotherapy. This result illustrates the importance of complete surgical resection. However, this does not mean that chemoradiotherapy is ineffective for MTTMJ. Whether adjuvant chemoradiotherapy improves prognosis could not be concluded from these results. Chemoradiotherapy was implemented empirically for highly malignant pathological types, suspicious or positive surgical margins and advanced stage tumours. It is obvious that the prognosis of these patients was worse than that of those who received surgery alone. At this point, our analysis is largely consistent with previous reports [7] [17].

The anatomy of the TMJ includes the condyle, fibrous capsule, disk, synovial membrane, fluid and adjacent ligaments [18]. The anatomical content may be the possible explanation for the TMJ harbouring a myriad of malignant tumours [19]. However, the pathological tumour type did not demonstrate a specific prognosis. The largest percentage of tumours originates from the condylar process, and tumours originating from the rest of the TMJ structure are much fewer. Among various pathological tumour types, sarcoma and osteosarcoma variants account for more than 95% of the total study population. We selected nine most commonly seen pathological tumour types with a sample size of greater than 15 for survival analysis. Despite other factors, malignant ameloblastoma had the highest survival rate, and unspecified malignant bone tumours showed the worst prognosis.

Generally, because MTTMJ’s signs and symptoms are similar to temporomandibular joint dysfunction, they may not receive sufficient attention [3]. Also, it is important to be aware of rare condition that could be misdiagnosed as a malignancy with the resulting unnecessary radical therapy. Therefore, the stomatologist must be alert, keeping in mind the occurrence of primary and metastatic tumours in the temporomandibular joint [5]. Like other solid malignant tumours in the head neck region, to deal with MTTMJ, early detection, early diagnosis and early treatment is fundamental. When clinical examination is suspicious, CT and MRI play an irreplaceable role in the early diagnosis and differential diagnosis of MTTMJ.

As a retrospective study, some limitations of this study and the SEER database should be acknowledged. Indeed, the SEER database provides the largest dataset for MTTMJ, which is one of its greatest advantages. However, the incompleteness of the data undermines its advantages. There are no data on clinical manifestations, and it is impossible to compare and discuss with previous reports. The exact orientation of the tumour remains unknown. However, it is certain that the majority of tumours originate from the condyle. Data on important variables, such as surgery types and TMJ reconstruction details, are incomplete. As superficial tumours, postoperative repair and reconstruction of the TMJ is more difficult than complete resection of the primary tumour. The quality of TMJ reconstruction has a great influence on the facial symmetry, function of the TMJ, occluding relation and postoperative quality of life. These factors are closely related to prognosis. Among the pathological types, squamous cell carcinoma accounted for a proportion of samples. The cervical lymph node metastasis rate of squamous cell carcinoma is relatively high. Whether neck dissection had been performed could not be determined in this cohort.

## Conclusions

For the first time, we attempted to conduct a retrospective study on the epidemiological characteristics, clinicopathologic features, treatment, survival and prognostic factors of TMJ malignancy with the largest sample size. The study results demonstrate that MTTMJ mostly occurred in white people and that the median age at diagnosis was 47 years. There was no significant morbidity or mortality difference by sex. The patient’s age and AJCC stage were independently associated with OS and DSS. Despite the limitations, our study results are an important reference for the future diagnosis, treatment and prognostic assessment of TMJ malignancy. As a retrospective study with lower level of evidence, our findings in MTTMJ management requires validation in further multicenter, longitudinal, prospective, large cohort studies.

## Data Availability

Study data was publicly available in the SEER database.

## Abbreviations

TMJ: Temporomandibular joint
MTTMJ: Malignant tumours of the temporomandibular joint
SEER: Surveillance, Epidemiology, and End Results
ICD-O-3: International Classification of Diseases for Oncology
AJCC: American Joint Committee on Cancer
SPSS: Statistical Package for the Social Sciences
OS: Overall survival
DSS: Disease-specific survival
